# Differentially Expressed Proteins Associated with Fusarium Head Blight Resistance in Wheat

**DOI:** 10.1371/journal.pone.0082079

**Published:** 2013-12-20

**Authors:** Xianghui Zhang, Jianming Fu, Yasuaki Hiromasa, Hongyu Pan, Guihua Bai

**Affiliations:** 1 Jinlin University, Changchun, Jilin, People's Republic of China; 2 United States Department of Agriculture/Agricultural Service, Hard Winter Wheat Genetics Research Unit, Kansas State University, Manhattan, Kansas, United States of America; 3 Department of Biochemistry, Kansas State University, Manhattan, Kansas, United States of America; 4 Department of Plant Pathology, Kansas State University, Manhattan, Kansas, United States of America; National Rice Research Center, United States of America

## Abstract

**Background:**

Fusarium head blight (FHB), mainly caused by *Fusarium graminearum*, substantially reduces wheat grain yield and quality worldwide. Proteins play important roles in defense against the fungal infection. This study characterized differentially expressed proteins between near-isogenic lines (NILs) contrasting in alleles of *Fhb1*, a major FHB resistance gene in wheat, to identify proteins underlining FHB resistance of *Fhb1*.

**Methods:**

The two-dimensional protein profiles were compared between the *Fusarium*-inoculated spikes of the two NILs collected 72 h after inoculation. The protein profiles of mock- and *Fusarium*-inoculated *Fhb1^+^*NIL were also compared to identify pathogen-responsive proteins.

**Results:**

Eight proteins were either induced or upregulated in inoculated *Fhb1^+^*NIL when compared with mock-inoculated *Fhb1^+^*NIL; nine proteins were either induced or upregulated in the *Fusarium*-inoculated *Fhb1^+^*NIL when compared with *Fusarium*-inoculated *Fhb1^−^*NIL. Proteins that were differentially expressed in the *Fhb1^+^*NIL, not in the *Fhb1^−^*NIL, after *Fusarium* inoculation included wheat proteins for defending fungal penetration, photosynthesis, energy metabolism, and detoxification.

**Conclusions:**

Coordinated expression of the identified proteins resulted in FHB resistance in *Fhb1^+^*NIL. The results provide insight into the pathway of *Fhb1*-mediated FHB resistance.

## Introduction

Wheat (*Triticum aestivum*) Fusarium head blight (FHB), mainly caused by *Fusarium graminearum*, is a destructive wheat disease in warm and humid regions worldwide [1], [2]. FHB causes premature spike death or blighting and substantially reduces grain yield and quality [1]. A recent FHB outbreak erupted in the major wheat-growing areas in China and caused yield losses from 10 to 100% in 2012 [X-H. Zhang, 2012, unpublished data]. In the U.S., FHB has spread south and is becoming more frequent and severe in the Great Plains. Infected grains are contaminated with mycotoxins that are harmful to human and animal health when they are used as food or feed [2]. The most common toxin associated with FHB is deoxynivalenol (DON); DON-contaminated wheat grains have undesirable end-use quality, thus low grain sale price.

Although certain cultural practices or timely application of fungicides can reduce FHB damage, the most economically effective and environmentally friendly approach to reducing the losses caused by this disease is to grow resistant cultivars [1]. To date, a number of germplasm lines from China, Europe, and the U.S. have been identified with a high level of FHB resistance [1], [3]. Among them, ‘Sumai3’ and its derivatives, such as ‘Ning7840’, showed the best resistance to FHB. *Fhb1* from Sumai3 has been used in breeding programs worldwide because it has shown the largest effect on FHB resistance identified so far [2]–[4]. Detailed wheat defense mechanisms against FHB infection, however, remain poorly characterized.

Plants may mobilize a variety of biochemical and molecular defenses to delay pathogen growth or resist pathogen infection [5]. An incompatible interaction between a host and a pathogen triggers defense responses through signaling pathways that can activate a broad series of defense responses to restrict pathogen growth or destroy the pathogen. These responses include hypersensitive reactions, deposition of cell wall reinforcing materials, and synthesis of a wide range of antimicrobial compounds such as pathogenesis-related (PR) proteins [6]. Several gene expression studies have been conducted in an attempt to understand the molecular mechanisms of interaction between cereal crops and *F. graminearum*. In barley, microarray analysis revealed that a majority of host gene transcripts were expressed in the intermediate infection stage by *F. graminearum* [7]. In wheat, Pritsch et al. [8] observed that the transcripts of defense response genes, peroxidase and PR-1 to -5, accumulated as early as 6 to 12 h after wheat spikes were inoculated with *F. graminearum*. Gottwald et al. [9] suggested that Jasmonate and ethylene dependent defense and suppression of fungal virulence factors are major mechanisms of FHB resistance in wheat. Lemmens et al [10] hypothesize that *Fhb1* resistance is due to a DON-glucosyl-transferase that detoxicify DON in Sumai3 or its derivatives; but several other more recent gene expression studies did not support the hypothesis [11]–[13]. Therefore, the genes involved in perceiving the pathogen attack signal and the gene expression cascade for FHB resistance remain to be elucidated.

The proteomic approach is a powerful tool to study mechanisms of plant resistance to fungal infection. An initial proteomic study on the interaction between *F. graminearum* and wheat was conducted to identify FHB infection response proteins by comparing protein profiles of *F. graminearum*- inoculated with mock-inoculated wheat spikelets of ‘Ning7840’, an *Fhb1* carrier, and gel-based proteomic analysis of the resistant cultivar revealed accumulation of plant proteins involved in oxidative stress, PR responses, and nitrogen metabolisms [14]. A further study revealed upregulation of proteins in the antioxidant and jasmonic acid-signaling pathway and PR responses and amino acid synthesis after 3 days of inoculation [15]. A similar study was done for an FHB-resistant Chinese landrace ‘Wangshuibai’ [16]. Protein profiles in these studies were compared between a mock- and *Fusarium*-inoculated cultivar, however, which provides information only on how a plant responds to pathogen attack, not how the plant resisted the pathogen infection. Because *Fhb1* has shown the largest effect on FHB resistance among FHB resistance genes reported to date, comparative analysis of protein profiles of near-isogenic lines (NILs) contrasting in *Fhb1* alleles should shed light on wheat resistance mechanisms to FHB. Only one study compared protein profiles between NILs that were developed from two backcrosses and the resistant NIL contains 89% of recurrent genome [11]. A resistant NIL with a higher proportion of recurrent genome will minimize background effect on the expression of the resistance gene. We have developed such a set of NILs by transferring the *Fhb1* resistance allele to a susceptible cultivar (‘Clark’) through backcrossing for seven times [17] and used the NILs to profile differentially expressed *Fhb1* related proteins.

## Materials and Methods

### Pathogen inoculum preparation

The pathogen inoculum was a field isolate of *F. graminearum* that originated in Kansas. Mung bean broth medium was used to grow *F. graminearum* conidia, and was made by boiling 40 g of mung beans in a 1-l flask for 10 min, then removing the beans by filtering the liquid through a piece of cheesecloth. About 100 ml of the broth in each 250-ml Erlenmeyer flask was autoclaved, inoculated with the mycelium of *F. graminearum* when the liquid was cooled, and then placed on a shaker running at 220 RPM for 4 days at 25°C to grow conidia. Conidial suspensions were diluted with autoclaved water to a final concentration of 100 spores/µl and stored at 4°C for inoculation.

### Plant materials and disease inoculation

Two NILs, NIL75 (*Fhb1^+^*NIL) and NIL98 (*Fhb1* ^−^NIL), were developed by backcrossing ‘Clark’ (a highly FHB susceptible parent) to ‘Ning 7840’ (*Fhb1* donor) seven times [17]. *Fhb1^+^*NIL contains less than 0.5% of donor genome. After seedlings of both lines were vernalized for 6 weeks at 4°C in a growth chamber, they were transplanted into 10.8-cm Dura-pots containing Metro-Mix 360 soil mix (Hummert Int, Earth City, MO) and grown in a greenhouse with 12 h supplemental light. For each treatment, 3 pots were transplanted with 5 plants per pot.

At anthesis, 10 µl of *F. graminearum* conidial suspension (100 spores/µl) was injected into a spike with a syringe. Mock inoculation used the same amount of mung bean broth served as a negative control. For each treatment, 9 plants in three pots were inoculated with 3 spikes per pot. All inoculated spikes were misted with distilled water and bagged after inoculation to maintain inoculated spikes at 100% relative humidity. Previous reports indicated that most proteins related to FHB resistance expressed at 72 h after inoculation [11], [15], therefore all inoculated and control spikes were harvested 72 h after inoculation. The harvested tissues were placed immediately into liquid nitrogen and then stored in a −80°C freezer until protein extraction.

The NILs were also evaluated for FHB resistance in a separated experiment using the same protocol as previously described. In each experiment, 5 pots per NIL with 5 plants per pot were inoculated with conidia at anthesis. FHB was scored at 18 days after inoculation and calculated as mean percentage of symptomatic spikelets per spike (PSS). The experiments were repeated twice.

### Protein extraction and quantification

Frozen mock- and *Fusarium*-inoculated spikes collected from each replication were weighed, transferred into a pre-chilled mortar, and ground into fine powder in liquid nitrogen. The powder was added with three times (g/ml) extraction buffer (50 Mm Tris-HCL, 2 mM EDTA, 10 mM β-mercaptoethanol, 10% glycerol (v/v), 1% protease inhibitor cocktail (v/v), mixed, and transferred into a 2.0-ml tube. The tube was centrifuged at 16,000 g for 20 min. The supernatant was transferred into a new tube for centrifuging again at 16,000 g for another 20 min. The supernatant in the new tube was aliquoted and frozen at −80°C. All the above procedures were operated at 4°C. Proteins were cleaned up using a kit from Bio-Rad Laboratories Ltd. (Hercules, CA), and the pellets were re-suspended in a rehydration buffer (7M urea, 2M thiourea, 1% ASB-14, 40 mM Tris, 0.001% bromophenol blue, 1% DTT, and 1% Bio-lyte buffer). The procedure for protein quantification followed Fu et al. [18]. Protease inhibitor cocktail for plant cell and tissue extracts was purchased from Sigma-Aldrich (Milwaukee, WI). All other reagents used for 2D-gel were purchased from Bio-Rad Laboratories Ltd.

### Isoelectric focusing (IEF) and SDS–PAGE

Isolated proteins from three biological replications of each treatment were pooled and approximately 120 ug of each protein sample was mixed with the rehydration buffer (Bio-Rad Laboratories Ltd.) to a total volume of 350 µl. All proteins were passively rehydrated for 16 h and absorbed into a 17-cm pH 3–10 (NL) Bio-Rad Ready Gel Strip according to the manufacturer's instruction (Bio-Rad Laboratories Ltd.). The IEF steps were 100 V for 1 h, 250 V for 2 h, 500 V for 2 h, 1,000 V for 1 h, 4,000 V for 1 h, 8,000 V for 1 h with a linear gradient, holding at 8,000 V until a total of at least 95,000 Vh was reached, then holding at 500 V. Before SDS-PAGE, the IEF strips were equilibrated in 5 ml equilibration buffer I [6 M urea, 2% (w/v) SDS, 0.05 M Tris–HCL (pH 8.8), 20% (v/v) glycerol, 2% (w/v) DTT] at ambient temperature for 15 min, then in the same volume of equilibration buffer II [6M urea, 2% (w/v) SDS, 0.05 M Tris–HCL (pH 8.8), 20% (v/v) glycerol, 2.5% (w/v) iodoacetamide] for another 15 min. For SDS-PAGE, the strips were positioned on top of the second dimension 12% (w/v) polyacrylamide gel and sealed with 1% (w/v) agarose gel. The gels were run at 100 V for 60 min followed by 200 V for 6 h on PROTEAN II XI Cell (Bio-Rad Laboratories Ltd.).

### Gel staining and image analysis

SDS-PAGE gels were stained using the colloidal CBB G250 staining method [19]. The stained gels were scanned on an EPSON 1680 scanner (EPSON, Long Beach, CA). Triplicate images from three independent gels (technical replications) for each treatment were obtained, and the normalized volumes of the three images were derived using Image Master 2D Platinum 6.0 DIGE software (GE Healthcare Biosciences, Pittsburgh, PA) and were averaged for quantification of each spot. Protein spots with at least 1.5-fold change in spot density and volume between infected and mock-inoculated samples or between inoculated NILs contrasting in *Fhb1* alleles were selected for further analysis. The selected protein spots were manually excised for sequencing.

### In gel digestion

Excised gel pieces were incubated in 100 µl of 50% acetonitrile at 30°C for 10 min. After de-staining, the gel pieces were shrunk by adding 50 µl of 100% acetonitrile for 10 min. After the solvent was discarded, the gel plugs were dried by a speed vacuum concentrator and incubated with 200 ng sequencing-grade trypsin (Trypsin Gold, Promega, Madison, WI) in 20 µl of 20-mM ammonium bicarbonate. Upon rehydration, the gel plugs were incubated with an additional 20 µl of 20-mM ammonium bicarbonate and 10% acetonitrile at 30°C for 17 h. Tryptic peptides were recovered from the gel plugs using 100 µl of 50% acetonitrile in 2% trifluoroacetic acid (TFA) at 30°C for 30 min. Extracted peptides were concentrated in a speed vacuum concentrator and added with 100 µl of 2% acetonitrile in 0.1% formic acid.

### Protein identification

Nano-HPLC was performed automatically using a micro-column switching device, Switchos (LC Packings, Amsterdam, the Netherlands), coupled to an autosampler, Famos (LC Packings), and a nanogradient generator, UltiMate Nano HPLC (LC Packings). Peptide solution (30 µl) was loaded to a C18 reversed-phase capillary column (75 µm ID×15 cm, PepMap: Dionex) in conjunction with an Acclaim C18 PepMap trapping column (300 µm ID×10 mm, Dionex). Peptides were separated by a nanoflow linear acetonitrile gradient using buffer A (0.1% formic acid, 2% acetonitrile) and buffer B (0.1% formic acid, 80% acetonitrile), starting from 5% to 60% buffer B over 45 min at a flow rate of 200 nl/min. The column was then washed in 95% buffer B for 5 min. System-control software Hystar 3.2 (Bruker Daltonics Inc., Billerica, MA) was used to control the entire process. The eluted peptides were injected into an HCT Ultra Ion Trap Mass Spectrometer (Bruker Daltonics Inc., Billerica, MA). The mass spectrometer was set up in a data-dependent MS/MS mode to acquire full scans (m/z acquisition range from 300 to 1500 Da). The four most intense peaks in any full scan were selected as precursor ions and fragmented by collision energy. MS/MS spectra were interpreted and peak lists were generated by DataAnalysis 3.4 and Biotools 3.0 software (Bruker Daltonics Inc., Billerica, MA).

### Bioinformatics

Peptide masses were compared to the NCBInr (April, 2012) database and *Triticum aestivum* EST database (downloaded in November 2012 from NCBI EST database) using MASCOT 2.3 (http://www.matrixscience.com) and Proteinscape 2 (Bruker Daltronics). The EST database included 858,408 EST sequences. The following parameters were used in all searches: maximum number of missed cleavages allowed was 2; mass tolerance was 1.2 Da for MS and 1.0 Da for MS/MS; and taxonomy was *Triticum aestivums*. Fixed modification was set on cystein with carbamidomethylation. Variable modification was done on methionine with oxidation and asparagine/glutamine with deamidation. The expectation values for accepting individual MS/MS spectra were 32 for protein database and 52 for EST database, which represent identity or extensive homology of probability lower than 0.05 (*P*<0.05). Peptides scoring less than 20 were rejected automatically to ensure that all protein identifications were reliable. Homology searches against matched EST sequences were conducted using the BLAST program (http://www.ncbi.nlm.nih.gov/).

## Results and Discussion

### Unique wheat materials for profiling wheat defense-related proteins

Although many studies have been done using gene expression assays [8], [9], [12], [20], a limited number of protein-profiling studies on FHB resistance have been conducted to date. In most of these protein studies, protein profiles were compared between pathogen-inoculated and mock-inoculated plants from a single genotype [14], [16], or from two unrelated genotypes that contrast in both disease resistance and genetic backgrounds [15]. In these cases, many differentially expressed proteins between two treatments could be basal defense proteins or due to difference in genetic backgrounds; thus, most of them may not be the proteins underlying plant resistance to the pathogen. Only in one study, *Fhb1* NILs containing 89% of recurrent genome were used [11]. In that study, *Fhb1* was derived from HC374 and might originate from either Wuhan (a unknown source of FHB resistance) from China or Nyubai from Japan. In this study, we developed a pair of NILs by backcrossing ‘Clark’ (a U.S. winter wheat cultivar) to ‘Ning7840’ (a *Fhb1* carrier derived from Sumai3) seven times to minimize the background difference between the NILs; thus, the selected NILs contrast in *Fhb1* alleles but share more than 99.5% recurrent genome and are ideal genetic materials for identifying proteins for FHB resistance related to *Fhb1*.

**Figure 1:**
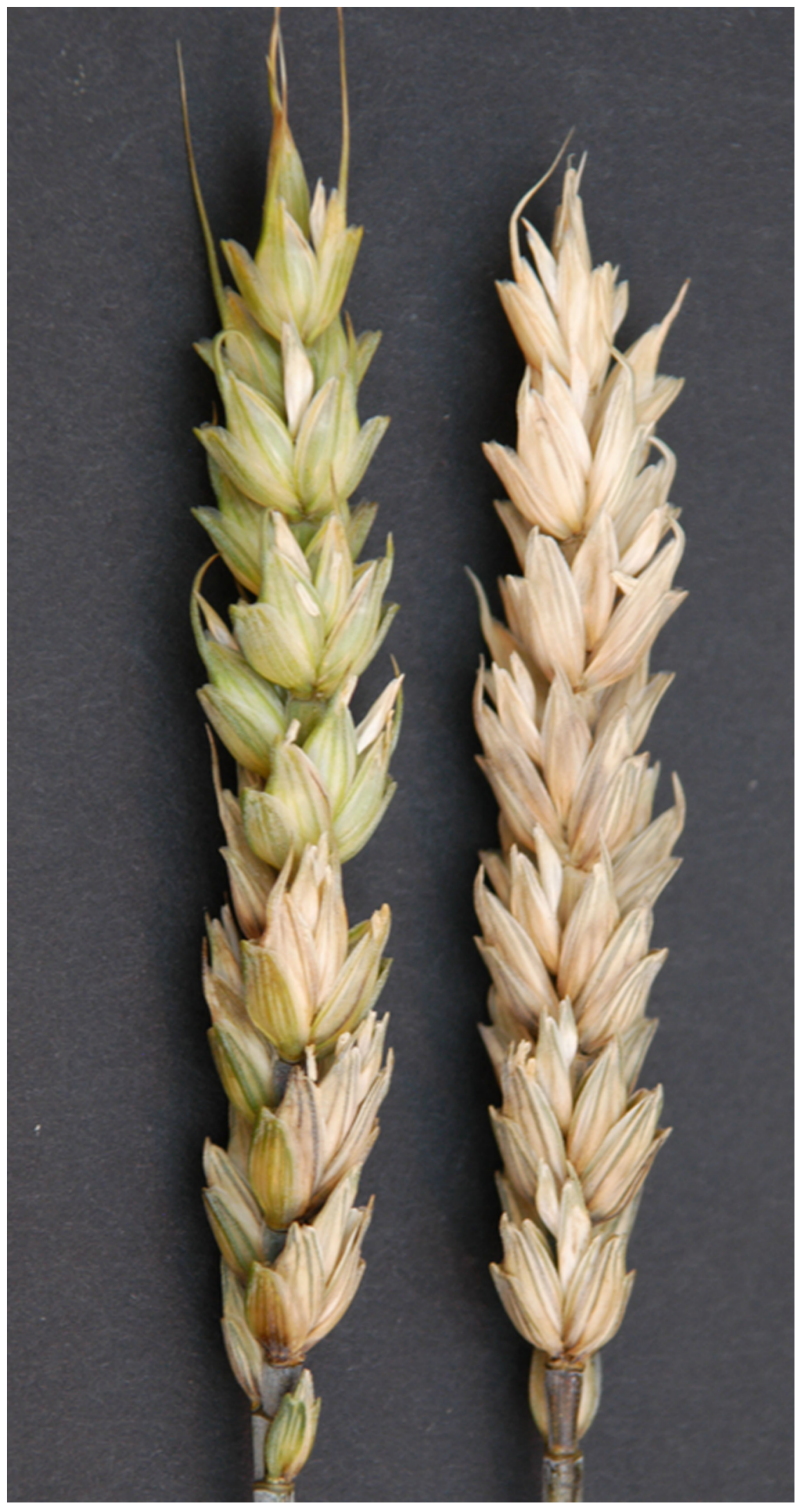
Wheat spikes of *Fhb1* ^+^NIL (NIL75, left spikes) and *Fhb1* ^−^NIL (NIL75, right spike) at 18th day after single spikelet inoculation with *F. graminearum*. *F. graminearum* infection did not spread or spread to only several uninoculated spikelets in *Fhb1* ^+^NIL, but entire spike *Fhb1* ^−^NIL was blighted.

To confirm the difference in FHB resistance between the two NILs, single-floret inoculation was conducted in the greenhouse. At 21 days after inoculation, the *Fhb1* ^+^NIL showed infection either in inoculated spikelets only or spreading to one to several uninoculated spikelets (Fig. 1); whereas the *Fhb1* ^−^NIL showed infection spread from the inoculated spikelet to most or all of uninoculated spikelets in the inoculated spikes and all infected spikelets were completely bleached. The mean PSS was 22.8% for the *Fhb1* ^+^NIL and 76.0% for the *Fhb1* ^−^NIL over three greenhouse experiments, showing a significant contrast (53%) in PSS between the two contrasting NILs. The differentially expressed proteins in the *Fhb1* ^+^NIL, not in the *Fhb1* ^−^NIL, after inoculation with *F. graminearum* are most likely related to FHB resistance regulated by *Fhb1*.

### Wheat proteins responsive to *F. graminearum* inoculation

**Figure 2:**
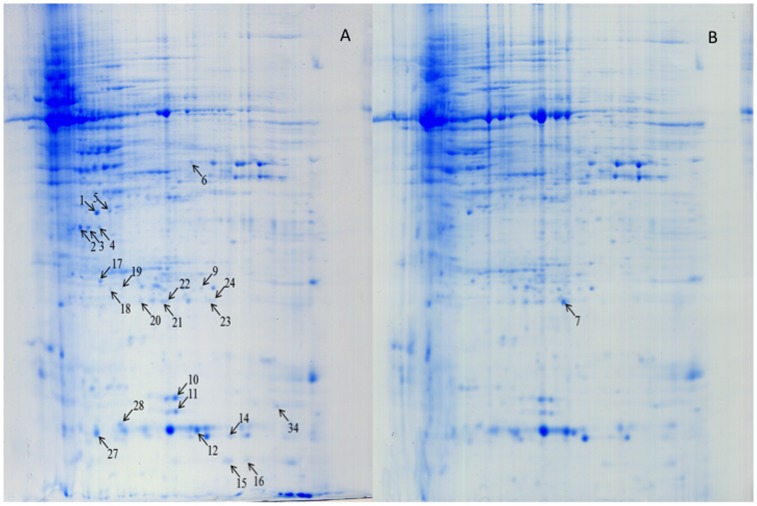
Protein profiles of wheat spikes of *Fhb1* ^+^NIL (NIL75) inoculated with *F. graminearum* (A) and mung bean broth (B) after 72 h.

**Figure 3:**
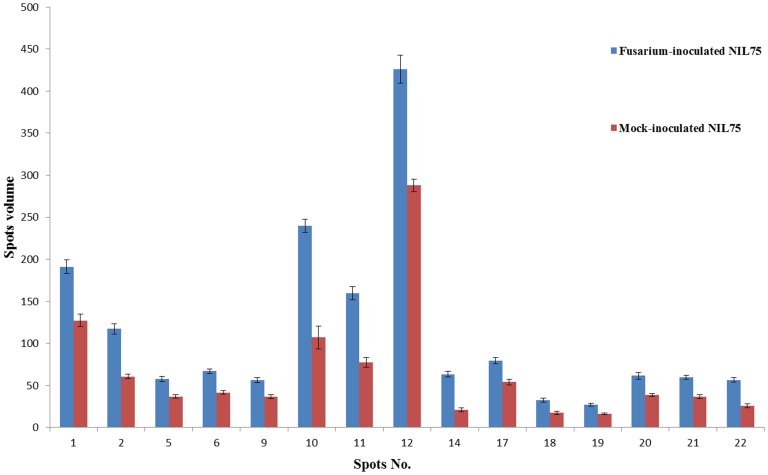
Histograms show the volume changes of 15 upregulated spots in *F. graminearum*-inoculated NIL75 (*Fhb1* ^+^NIL) and mock-inoculated NIL75.

Protein profiles of spikes collected 72 h after inoculation with *F. graminearum* were compared with 72 h mock-inoculated spikes. Four proteins from seven spots were induced (qualitative difference) by *F. graminearum* inoculation, and they presented only in *F. graminearum*-inoculated *Fhb1^+^*NIL spikes, not in the mock-inoculated *Fhb1^+^*NIL spikes (Fig. 2). These proteins were chloroplast oxygen-evolving enhancer protein 1 (OEE1, spots 3 and 4), PR-4 (spots 15 and 16), OEE 2 (spots 23 and 24), and single stranded nucleic acid binding (SSB) protein (spot 28) (Fig. 2; Table 1). Eleven proteins from 17 spots were significantly upregulated (quantitative difference) in *Fhb1^+^*NIL spikes after *F. graminearum* inoculation when compared with the mock-inoculated spikes (Fig. 3); they were Rossmann-fold NAD(P)(+)-binding proteins (Fig. 2, spots 1 and 5), chloroplast OEE1 (spot 2), glyceraldehyde-3-phosphate dehydrogenase (GAPDH, spot 6), superoxide dismutase (SOD, spots 9, 10 and 11), nucleoside diphosphate kinase (NDPK, spots 12 and 34), 20 kDa chaperonin (spot 17), OEE 2 (spots 18, 19, 20, 21), SSB protein (spot 27), and an unknown protein (spot 22) (Fig. 2; Table 1). These proteins, either pathogen-induced or upregulated, were mainly involved in stress response, PR response, resistance to fungal penetration, plant photosynthesis, and energy metabolism. Some, such as GAPDH, SOD [14], [15], and EEO [16], have been reported previously as *Fusarium*-responsive proteins. Only two protein spots were upregulated in *Fhb1^−^*NIL spikes after *F. graminearum* inoculation when compared with the mock-inoculated spikes of the same NIL (Data not shown). The results suggest that *Fhb1* ^+^NIL had a majority of genes that were either induced or upregulated in response to inoculation of *F. graminearum* than *Fhb1* ^−^NIL, which agrees with previous reports [11], [15], [16], [21].

**Table 1:** Differentially expressed proteins identified by ESI–MS/MS.

Spot No.	gi number	description/function	Frame	MW	pI	Mascot Protein Score	unique peptides	coverage
1	gi|383674360|gb|HX134554.1|HX134554	Rossmann-fold NAD(P)(+)-binding proteins*	3	58.9	6.9	529	7	14*
	gi|383674331|gb|HX134525.1|HX134525	Rossmann-fold NAD(P)(+)-binding proteins*	4	70.3	7	262	3	9*
2	gi|383633628|gb|HX052023.1|HX052023	Photosystem II oxygen-evolving enhancer protein 1*	2	61.1	6.9	809	14	60*
	gi|73912433	Aspartic proteinase [Triticum aestivum]		54.3	5	289	5	14
3	gi|383633628|gb|HX052023.1|HX052023	Photosystem II oxygen-evolving enhancer protein 1*	2	61.1	6.9	664	12	53*
	gi|73912433	Aspartic proteinase [Triticum aestivum]		54.3	5	163	3	10
4	gi|383633628|gb|HX052023.1|HX052023	Photosystem II oxygen-evolving enhancer protein 1*	2	61.1	6.9	693	11	53*
5	gi|383741321|gb|HX179787.1|HX179787	Rossmann-fold NAD(P)(+)-binding proteins*	2	57.4	7	433	6	46*
	gi|383674331|gb|HX134525.1|HX134525	Rossmann-fold NAD(P)(+)-binding proteins	4	70.3	7	274	7	40*
6	gi|253783729	Glyceraldehyde-3-phosphate dehydrogenase		36.5	6.8	309	5	18
	gi|383741321|gb|HX179787.1|HX179787	Phenylcoumaran benzylic ether reductase (PCBER) like*	2	57.4	7	284	4	33
7	gi|131394	Oxygen-evolving enhancer protein 2, chloroplastic		27.3	9.5	541	7	37
9	gi|125663927	Manganese superoxide dismutase		19.3	6.9	266	5	40
10	gi|226897529	Superoxide dismutase		15.1	5.7	244	5	29
11	gi|226897529	Superoxide dismutase		15.1	5.7	192	3	28
12	gi|383599548|gb|HX251834.1|HX251834	Nucleoside diphosphate kinase Group I (NDPk_I)-like*	4	40.8	7	436	7	45
	gi|383670986|gb|HX111086.1|HX111086	Ribulose bisphosphate carboxylase/oxygenase (Rubisco), small subunit*	2	56.5	7	261	7	20
14	gi|82619	Ribulose-bisphosphate carboxylase (EC 4.1.1.39) small chain precursor		15.3	9.9	131	4	21
15	gi|6048567	Pathogenesis-related protein 4		13.1	6.3	208	2	30
16	gi|6048569	Pathogenesis-related protein 4		13.1	7.8	146	3	28
17	gi|383595701|gb|HX164281.1|HX164281	20 kDa chaperonin, chloroplastic-like*	4	63.9	7	847	13	57
18	gi|131394	Oxygen-evolving enhancer protein 2, chloroplastic		27.3	9.5	445	6	26
19	gi|383675855|gb|HX136948.1|HX136948	Oxygen-evolving enhancer protein 2*	2	61.9	7	372	6	27
20	gi|131394	Oxygen-evolving enhancer protein 2, chloroplastic		27.3	9.5	354	5	24
21	gi|131394	Oxygen-evolving enhancer protein 2, chloroplastic		27.3	9.5	114	3	15
22	n.d.							
23	gi|131394	Oxygen-evolving enhancer protein 2, chloroplastic		27.3	9.5	393	5	23
24	gi|131394	Oxygen-evolving enhancer protein 2, chloroplastic		27.3	9.5	531	7	26
27	gi|974605	Single-stranded nucleic acid binding protein		16.2	5	372	6	81
	gi|11990893	Ribulose-1,5-bisphosphate carboxylase/oxygenase small subunit		19.5	9.8	169	4	26
28	gi|974605	Single-stranded nucleic acid binding protein		16.2	5	180	3	35
	gi|11990897	Ribulose-1,5-bisphosphate carboxylase/oxygenase small subunit		19.4	9.7	140	4	20
34	gi|383743921|gb|HX172565.1|HX172565	Nucleoside diphosphate kinase Group I (NDPk_I)-like*	5	58.6	7	293	5	25
46	gi|383608201|gb|HX252082.1|HX252082	WD40 domain*	2	53.7	7	692	10	59
	gi|295917894	Beta-cyanoalanine synthase		40.2	6.4	266	5	18
47	gi|18146825	Chitinase 1		27.1	9.6	778	10	66
48	gi|1568639	Cu/Zn superoxide dismutase		20.3	5.3	263	4	31
	gi|196051131	Pathogenesis related protein 10		17.1	5.1	169	4	34
49	gi|383739814|gb|HX143464.1|HX143464	Nucleoside diphosphate kinase Group I (NDPk_I)-like*	4	45.9	7	177	3	16
50	gi|1572627	Cu/Zn superoxide dismutase		20.2	5.3	235	3	37
51	gi|1572627	Cu/Zn superoxide dismutase		20.2	5.3	144	3	37
52	gi|383606411|gb|HX251502.1|HX251502	Actin depolymerisation factor/cofilin -like*	6	54.7	6.9	248	4	41

* indicates that matched sequence was found in the EST and its function was the same as indicated by BLAST search.

### Wheat proteins associated with FHB resistance

**Figure 4:**
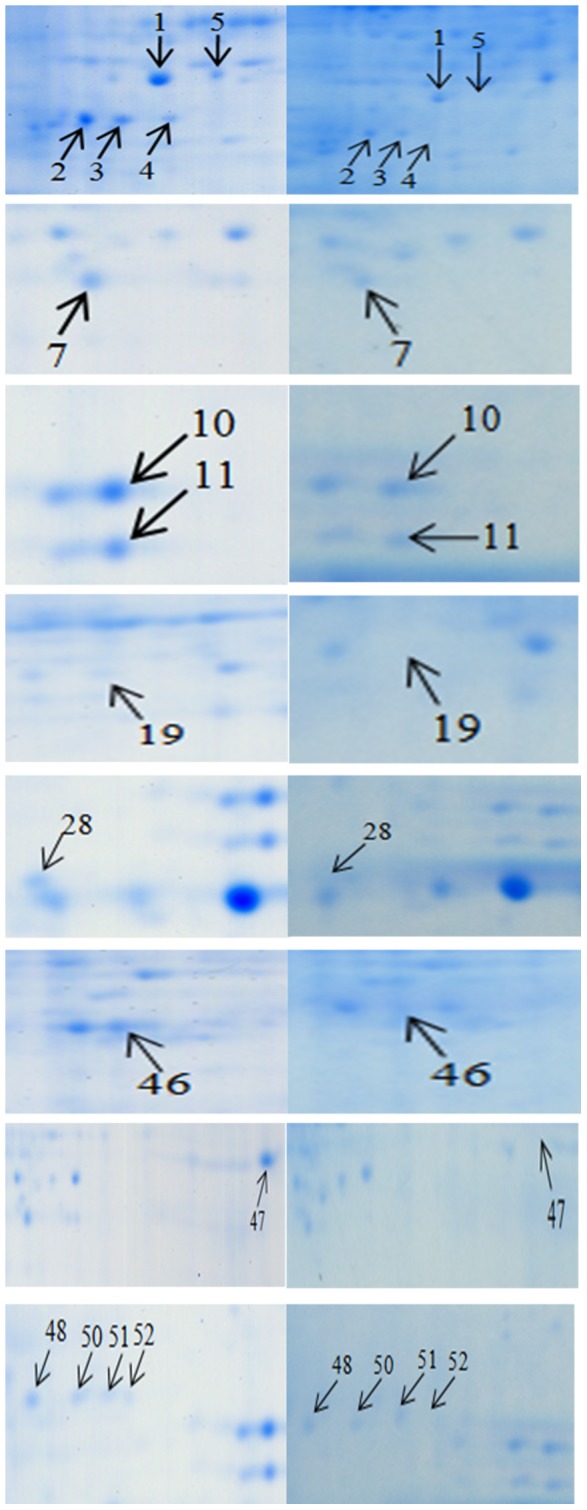
Enlarged partial gel images showing differentially expressed protein spots between inoculated resistant line *Fhb1* ^+^NIL (NIL75) and inoculated susceptible line *Fhb1* ^−^NIL (NIL98).

**Figure 5:**
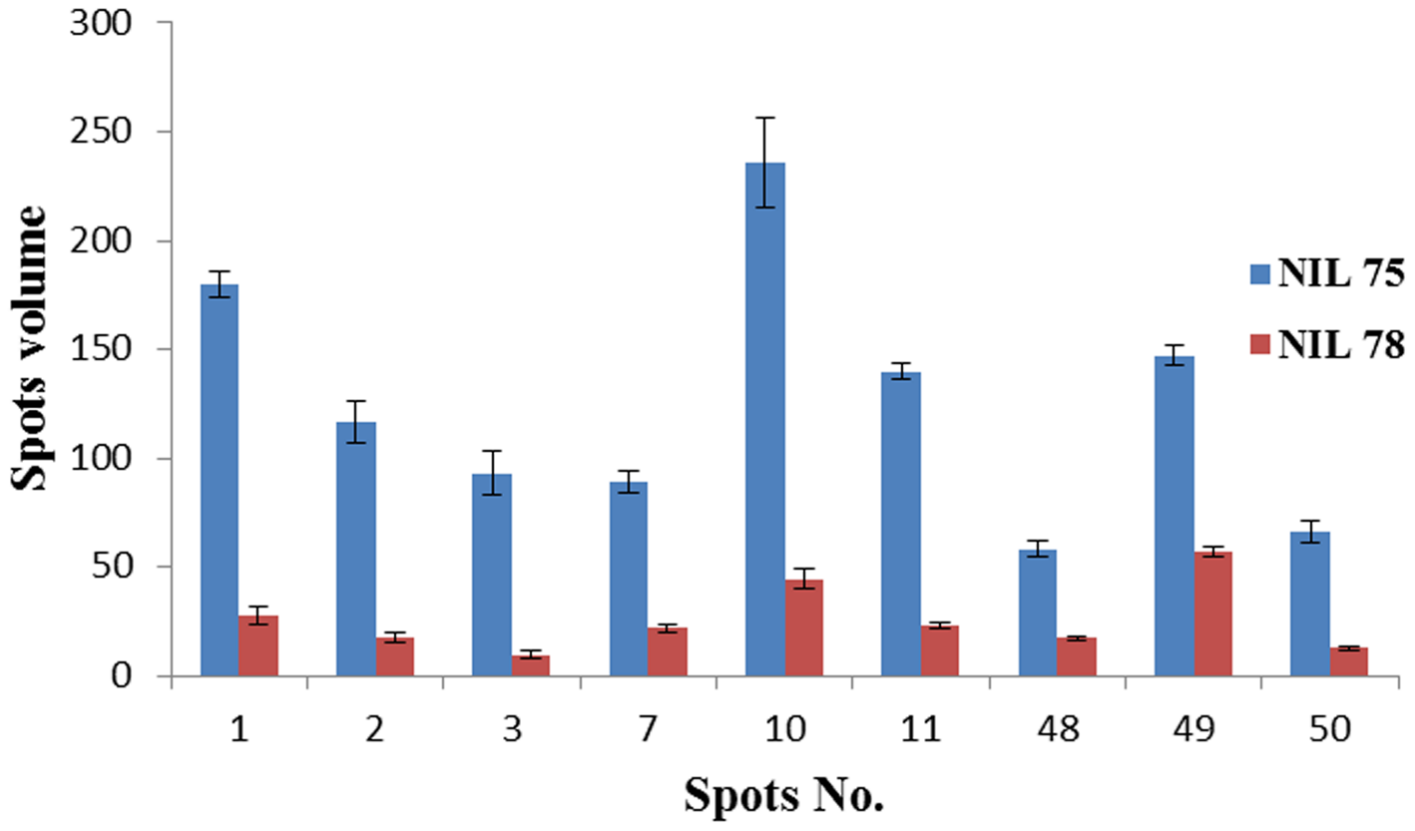
Histograms show the volume changes of 9 upregulated protein spots in the resistant NIL75 (*Fhb1* ^+^NIL) and the susceptible NIL98 (*Fhb1* ^−^NIL) after inoculation with *F. graminearum*.

To identify wheat proteins associated with resistance to *F. graminearum*, the protein profiles of *Fhb1^+^NIL* and *Fhb1^−^* NILs were compared after inoculation with *F. graminearum*. Fig. 4 presents representative gel images that were selected from three independent experiments showing differentially expressed proteins between the two inoculated NILs. Eight protein spots presented only in *Fhb1^+^*NIL, but not in the *Fhb1^−^*NIL after inoculated with *F. graminearum*; they were chloroplast OEE1 (spot 4) and OEE 2 (spot 19), Rossmann-fold NAD(P)(+)-binding proteins (spot 5), single-stranded nucleic acid binding protein (spot 28), beta-cyanoalanine synthase (CAS, spot 46), chitinase (spot 47), Cu/Zn SOD (spot 51), and actin depolymerisation factor (ADF)/cofilin-like (spot 52) (Fig. 4; Table 1). Nine protein spots showed significantly higher levels of expression in *Fhb1^+^*NIL than in *Fhb1^−^*NIL (Fig. 5): Rossmann-fold NAD(P)(+)-binding proteins (spot 1), chloroplast OEE1 (spots 2 and 3), OEE2 (spot 7), SODs (spots 10, 11 and 50), PR-10 (spot 48), and NDPKI-like protein (spot 49). Most of these differentially expressed proteins in *Fhb1* ^+^NIL were induced by *F. graminearum*, except CAS, chitinase, and ADF/cofilin. These proteins are mainly for oxidative stress responses, resistance to fungal penetration, plant photosynthesis, plant detoxification, etc. Some proteins such as SOD and chitinase have been reported previously as *Fusarium*-responsive proteins or resistance related proteins [11], [14], [15], [16], [21], but most are newly identified proteins related to FHB resistance in this study including an ADF/cofilin protein, CAS, NAD(P)(+)-binding protein, and NDPKI-like protein.

Resistance to pathogen penetration at the plant cell surface by formation of cell wall apposition (CWA), a physical and chemical barrier to cell penetration by a pathogen, is a key mechanism for plants. The actin cytoskeleton plays an important role in formation of CWA [22], and actin-binding proteins, such as ADF/cofilins, regulate the dynamic behavior of actin filaments during forming CWA. Actin dynamics demonstrated a role in the activation of gene-for-gene resistance of *Arabidopsis thaliana* to *Pseudomonas syringae* pv tomato [23]; abiotic stresses also induced significant expression of ADF/cofilins in cereal plants [24]. Thus, ADF/cofilins might be important proteins to protect plants against biotic and abiotic stresses. A high level of ADF/cofilin protein presented in only *Fhb1^+^*NIL suggests that CWA formation is an important initial step for wheat resistance to *Fusarium* early penetration in cell walls of spikelets in resistant wheat.

Reactive oxygen species (ROS) such as H_2_O_2_ also play an important role in plant-pathogen interaction [25]. Pathogen-induced H_2_O_2_ is required for peroxidase-dependent lignification that hinders the penetration of a pathogen [26], [27]. H_2_O_2_ is very important for resistance to fungal basal penetration because enzymatic removal of H_2_O_2_ enhances the fungal penetration on leaf epidermal cells [26]. H_2_O_2_ is also required for protein cross-linking in cell wall at the site of pathogen contact to produce a stress; the host cells' generation of an oxidative burst also stresses the pathogen [26], [27], [28]. The cross-linking of proline-rich proteins in the cell wall makes plant cells more resistant to cell wall–degrading enzymes produced by a pathogen and may entrap the fungal penetration peg in a CWA [28]. In the barley–*B. graminis* interaction system, the presence of H_2_O_2_ in CWAs can be used as a biochemical marker to identify nonpenetrated cells [29]. Because H_2_O_2_ is membrane-permeable, it may also act as a diffusible signal that leads to systemic acquired resistance [27]; thus, H_2_O_2_ might be a potent messenger in cell wall–associated defense. In plant cells, SOD can rapidly convert the O^2−^ to H_2_O_2_ that accumulates at the site of pathogen contact where CWA is formed, which restricts pathogen movement and reproduction, and prevents the spread of the pathogen to other parts of the plants [27], [30]. In this study, four spots of SODs were induced or upregulated in the inoculated *Fhb1^+^*NIL, not in the inoculated *Fhb1^−^*NIL (Fig. 4; Table 1); therefore, the SODs played a critical role in resistance to FHB penetration by strengthening cell walls. Several other studies also presented evidences to support that cell wall thickening is a major mechanism of FHB resistance [11], [22], [16].

Plants may actively defend against pathogen infection by producing enzymes that digest fungal cell walls to stop fungal penetration. Because all true fungi contain chitin as a primary structural component of their cell walls, the chitinase family of PR proteins is of particular importance [31]. Chitin in fungal cell walls can be hydrolyzed by chitinases into smaller oligomers or monomers [31], [32], so chitinases are considered to play a major role during plant–fungus pathogenic interactions [33]–[35]. Chitinase was reported to be upregulated in FHB-resistant wheat ‘Ning7840’ [15], [20]. Transgenic plants that overexpressed chitinases exhibited enhanced resistance to pathogens [36], [37]. Transgenic wheat that overexpressed a barley class II chitinase gene significantly increased Type II FHB resistance [37]. In this study, differentially expressed chitinase (Fig. 4, spots 47 and 48) that presented only in the *Fhb1^+^*NIL provides another line of evidence that the degradation of fungal cell wells by chitinases enhances FHB resistance in wheat.

Oxygen-evolving enhancer proteins (OEEs), consisting of three subunits [OEE1 (33 kDa), OEE2 (23 kDa), and OEE3 (16 kDa)], are nuclear-encoded chloroplast proteins and are peripherally bound to photosystem II (PSII) on the luminal side of the thylakoid membrane [38]. Photosynthetic oxygen evolution requires the interaction of several different yet closely coupled biochemical reactions. The light-capturing and charge-separating capacities of PSII must work in close cooperation with an oxygen-evolving complex capable of utilizing this oxidizing power to split water into oxygen and hydrogen. Electrons stripped from water during this reaction are funneled back into photochemical reaction center II, then transported through the electron transport chain to photosystem I, eventually to be used for the reduction of NADP [39]. Wang et al. [16] found that OEE2 of PSII was upregulated in FHB resistant cultivar ‘Wangshuibai’ after inoculation with *F. graminearum*. In barley, PSII oxygen-evolving complex protein 2 precursor was expressed in response to *F. graminearum* [40]. In the current study, both OEE1 (Fig. 4, protein spot 4) and OEE2 (spot 19) were detected in the *F. graminearum*-inoculated *Fhb1^+^*NIL, but not in the *Fhb1^−^*NIL, suggesting that the two OEEs played an important role in maintaining PSII activity when wheat was inoculated with *F. graminearum*. Mizobuchi and Yamamoto [41] demonstrated that OEE1 was essential for oxygen evolving activity and PSII stability. In wheat FHB, the most obvious visual disease symptom on the spike of a susceptible plant infected by *F. graminearum* starts with chlorosis to bleached spikes; thus, photosynthesis in the infected spikes is significantly reduced or stopped completely in a susceptible genotype [42]. Therefore, the recovery or turnover of OEEs in the inoculated FHB-resistant spikes may be attributed to maintaining the capacity of PSII for enhanced photosynthesis after *F. graminearum* attacks.

A higher level of NAD(P)(+)-binding proteins detected in inoculated *Fhb1* ^+^NIL than in *Fhb1* ^−^NIL also supported the idea that enhanced photosynthesis is related to FHB resistance. NAD(P)(+)-binding proteins bind nicotinamide dinucleotide (NAD) to catalyze reactions central to energy production, storage, and transfer. These reactions are essential to nearly all core metabolic pathways including photosynthesis [43]. Glyceradehyde-3-phosphate dehydrogenase (GAPDH) is a NAD(P)(+)-binding protein [43] and has been reported to be involved in photosynthetic metabolism and responses to abiotic [44], [45] and biotic stresses [46].

Nucleoside diphosphate kinases (NDPKs) are primary metabolic enzymes that maintain the balance between cellular ATP and other nucleoside triphosphates and play regulatory roles in response to multiple stresses [47]. In rice, *NDPK1* was reported to be involved in the defense against bacterial infection [43]. *NDPK* transcript was upregulated in response to wounding in tomato [48], and transgenic *Arabidopsis* that overexpressed *AtNDK1* exhibited tolerance to paraquat (N,N′-dimethyl-4,4′-bipyridinium dichloride), suggesting a role for *AtNDK1* in ROS response [49]. In this study, *F. graminearum* induced a higher level of NDPK1 expression in the *Fusarium*-inoculated *Fhb1^+^*NIL than *Fhb1^−^*NIL, which suggests that NDPK1 protein may be involved in defense against FHB infection in wheat.

Many biotic and abiotic stress conditions can induce ethylene production in plants, while HCN is a byproduct in the ethylene pathway [50]. HCN is extremely toxic to plant cells, but beta-cyanoalanine synthase (CAS) can rapidly detoxify HCN and recycle the reduced nitrogen of cyanide for amino acid synthesis [51]. Cell wall protein fractions from pathogens induced significant ethylene production in plants, and trace amounts of ethylene can elicit many physiological responses [50], [52], [53]; thus, the induced expression of CAS detoxified HCN resulting from the elevated level of ethylene production in cell wall protein–treated plants and the high level of CAS activity were caused by the general response to ethylene production. A previous study reported that wheat spikes challenged with cell wall proteins from *Pythium oligandrum* had a significantly reduced number of infected spikelets compared with the control after *F. graminearum* inoculation and demonstrated that CAS induced by fungal elicitors reduced the level of *Fusarium* infection in wheat [54]. Several proteins in ethylene signal pathway have been associated with FHB resistance [9], [21], [55]. In this study, a high level of CAS (spot 46) (Fig. 4; Table 1) was detected only in the *Fhb1* ^+^NIL, not in the *Fhb1* ^−^NIL, indicating the important role of CAS and ethylene signaling in preventing FHB spread within a spike after *Fusarium* infection in wheat.

Single stranded nucleic acid binding (SSB) protein is essential for DNA replication and repair in nearly all organisms, so it is crucial to genome maintenance [56]. SSB proteins bind with high affinity and specificity to ssDNA intermediates to protect them from degradation and destabilize inhibitory secondary structures within the ssDNA, and they regulate the activities of other proteins by direct binding to bring them to their sites of action on DNA [56]. In Arabidopsis, organellar single-stranded DNA binding protein 1 was shown to be required for mtDNA stability [57]. In this study, a significantly higher level of SSB protein was detected in inoculated *Fhb1^+^*NIL than in *Fhb1^−^*NIL, suggesting the SSB protein may be actively involved in protection of ssDNA in mitochondrial or chloroplast from degradation in FHB-resistant genotypes.

## Conclusions

Comparisons of protein expression profiles between the unique pair of NILs contrasting in *Fhb1* alleles identified nine types of either induced or upregulated proteins that were associated with wheat FHB resistance in the *Fhb1* ^+^NIL. These differentially expressed proteins may be involved in complicated processes to defend against fungal infection in FHB-resistant genotypes by degrading fungal cell walls and strengthening plant cell walls at the site of pathogen contact to hinder pathogen penetration; detoxifying toxic cyanide in the ethylene pathway; and maintaining photosynthesis and energy metabolism. Although the *Fhb1* was previously located on chromosome 3BS [4], whether the genes encoding these FHB resistance-related proteins are located on the same chromosome remains unknown. It is possible that *Fhb1* gene(s) on the chromosome 3BS trans-regulate the expression of some of these genes in downstream to provide FHB resistance; therefore, further cloning of *Fhb1* may elucidate the functions of *Fhb1* in the wheat FHB system.
